# Genetic heterogeneity in leiomyomas of deep soft tissue

**DOI:** 10.18632/oncotarget.17953

**Published:** 2017-05-17

**Authors:** Ioannis Panagopoulos, Ludmila Gorunova, Marta Brunetti, Antonio Agostini, Hege Kilen Andersen, Ingvild Lobmaier, Bodil Bjerkehagen, Sverre Heim

**Affiliations:** ^1^ Section for Cancer Cytogenetics, Institute for Cancer Genetics and Informatics, The Norwegian Radium Hospital, Oslo University Hospital, Oslo, Norway; ^2^ Centre for Cancer Biomedicine, Faculty of Medicine, University of Oslo, Oslo, Norway; ^3^ Department of Pathology, The Norwegian Radium Hospital, Oslo University Hospital, Oslo, Norway; ^4^ Faculty of Medicine, University of Oslo, Oslo, Norway

**Keywords:** leiomyoma of deep soft tissue, cytogenetics, HMGA2, PLAG1

## Abstract

Leiomyoma of deep soft tissue is a rare type of benign smooth muscle tumor that mostly occurs in the retroperitoneum or abdominal cavity of women, and about which very little genetic information exists. In the present study, eight leiomyomas of deep soft tissue were genetically analyzed. G-banding showed that three tumors carried rearrangements of the long arm of chromosome 12, three others had 8q rearrangements, the 7^th^ tumor had deletion of the long arm of chromosome 7, del(7)(q22), and the 8^th^ had aberrations of chromosome bands 3q21∼23 and 11q21∼22. The target genes of the 12q and 8q aberrations were *HMGA2* and *PLAG1*, respectively. In the leiomyomas with 12q rearrangements, both *HMGA2* and *PLAG1* were expressed whereas in the tumors with 8q aberrations, only *PLAG1* was expressed. In the cases without 12q or 8q aberrations, the expression of *HMGA2* was very low and *PLAG1* was expressed only in the case with del(7)(q22). All eight leiomyomas of deep soft tissue expressed *MED12* but none of them had mutation in exon 2 of that gene. In two tumors with 12q rearrangements, *RPSAP52* on 12q14.3 was fused with non-coding RNA (accession number XR_944195) from 14q32.2 or *ZFP36L1* from14q24.1. In a tumor with inv(12), exon 3 of *HMGA2* was fused to a sequence in intron 1 of the *CRADD* gene from 12q22. The present data together with those of our two previous studies in which the fusions *KAT6B-KANSL1* and *EWSR1-PBX3* were described in two retroperitoneal leiomyomas carrying a t(10;17)(q22;q21) and a t(9;22)(q33;q12) translocation, respectively, show that leiomyomas of deep soft tissue are genetically heterogenous but have marked similarities to uterine leiomyomas.

## INTRODUCTION

According to the 2013 edition of “WHO classification of tumours of soft tissue and bone”, leiomyoma of deep soft tissue is a “rare type of leiomyoma that occurs in deep soft tissue in the retroperitoneum or abdominal cavity, mostly in women” [1]. Though macroscopically always outside and distinct from the uterus, they have pathological and histological features similar to those of uterine leiomyomas, including common hyaline fibrosis, alternating myxoid change or trabecular patterns, and positivity for estrogen and progesterone receptors [2–4]. The cytogenetics and molecular genetics of retroperitoneal leiomyomas are largely unexplored and in the 2013 edition of “WHO classification of tumours of soft tissue and bone,” no genetic information on these tumors can be found [1]. In 2014, mutations in exon 2 of the *MED12* gene were reported in 34% of leiomyomas/leiomyomatoses of pelvic/retroperitoneal sites [5]. The authors concluded that “smooth muscle tumors in pelvic/retroperitoneal sites are subject to the same mutational changes as those of uterine myometrium, and [that] these mutations may precede the gross or histological development of a leiomyoma” [5].

Recently, we reported the first two cytogenetically analyzed retroperitoneal leiomyomas [6, 7]. In the first case, the tumor cells carried a t(10;17)(q22;q21) as the sole karyotypic aberration, the molecular consequence of which was fusion of the *KAT6B* gene (also known as *MORF* and *MYST4*) on 10q22 with the *KANSL1* gene (official full name: KAT8 regulatory NSL complex subunit 1) from 17q21 [6]. In the second case, the tumor cells had a t(9;22)(q33;q12) resulting in a fusion gene consisting of parts of *EWSR1* (from 22q12.2) and *PBX3* (from 9q33.3) [7]. These two studies showed that retroperitoneal leiomyomas may be characterized by fusion genes coding for chimeric proteins. However, the finding of different fusions indicated genetic heterogeneity and that various pathways could lead to retroperitoneal leiomyomagenesis.

We present here the genetic analysis of eight leiomyomas of deep soft tissue providing support for the conclusion that genetic heterogeneity, similar to the heterogeneity seen in their uterine counterparts, is a feature of these tumors.

## RESULTS

### Patients

**Figure 1:**
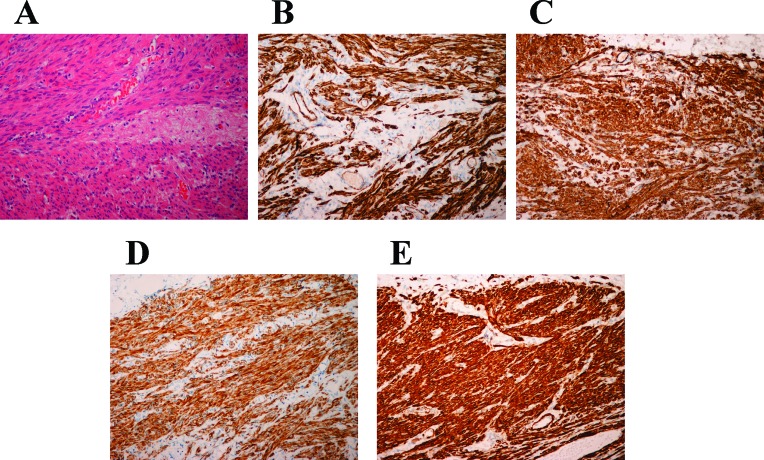
Histological examination of the leiomyoma of deep soft tissue of case 4 **A**. H&E-stained slide. **B**. Immunoexpression of actin. **C**. Immunoexpression of SMA. **D**. Immunoexpression of desmin. **E**. Immunoexpression of L-caldesmon. Magnification for all slides is x20.

All patients were females from 43 to 73 years old with a median age of 54. There was no history of uterine leiomyoma/hysterectomy (no information was available for case 5, Table 1). The tumor was single mass in all patients. The tumors were positive for estrogen receptor and progesterone receptor (no information was available for case 6). Table 1 shows the patients' gender, age, diagnosis, location and largest diameter of the tumors, and the results of immunohistochemical examinations using actin, aortic smooth muscle actin (SMA), desmin, L-caldesmon, estrogen receptor, and progesterone receptor. Figure 1 shows an H&E-stained section from case 4 as well as the immunoexpression of actin, SMA, desmin, and L-caldesmon in the same tumor.

**Table 1:** Clinicopathological data, karyotypes, interphase FISH results, and expressions of the *HMGA2* and *PLAG1* genes in eight leiomyomas of deep soft tissue

Case	Sex/Age	Location	Largest diameter (cm)	Immunohistochemistry-positive staining	Group	Karyotype	Interphase FISH rearrangements	Expression of *HMGA2*^a^	Expression of *PLAG1*^a^
1	F/53	retroperitoneum	11	Actin, SMA, desmin, estrogen receptor, progesterone receptor	1	44∼45,XX,der(12)del(12)(q13q21)ins(12;?)(q13;?),del(14)(q22),-21,-22[cp11]	HMGA2 (73 %)	5.31 ± 0.19	0.62 ± 0.06
2	F/47	preperitoneal adipose tissue	7	Actin, desmin, estrogen receptor, progesterone receptor	1	45∼46,XX,inv(12)(p11q15)[cp12]/46,XX[2]	HMGA2 (43 %)	0.78 ± 0.06	0.12 ± 0.01
3	F/68	preperitoneal	6	Actin, SMA, desmin, L-caldesmon, estrogen receptor, progesterone receptor	1	46,XX,t(3;13)(p21;p13),del(5)(q31q33),t(12;14)(q15;q24)[5]/46,idem,del(9)(q22)[10]	HMGA2 (98 %)	0.48 ± 0.09	0.42 ± 0.02
4	F/53	ovary/peritoneum	7	Actin, SMA, desmin, L-caldesmon, estrogen receptor, progesterone receptor	2	46,XX,ins(8)(p23q12q22)[30]	PLAG1 (75 %)	0.00	5.85 ± 0.60
5	F/43	abdominal wall/groin muscles	9	SMA, desmin, estrogen receptor, progesterone receptor	2	46,XX,t(8;14)(q13;q24)[15]	PLAG1 (86 %)	Not done	Not done
6	F/69	abdomen	11.5	Actin, SMA, desmin	2	46,XX,t(8;19)(q12;q13)[12]/46,XX[3]	Not done	0.00	0.42 ± 0.08
7	F/73	retroperitoneal/ left kidney	9.5	SMA, desmin, estrogen receptor, progesterone receptor	3	45∼46,XX,del(7)(q22)[cp6]/45∼46,idem,?del(14)(q24)[cp6]	Not done	0.02 ± 0.00	0.39 ± 0.02
8	F/56	abdominal wall	6.5	Estrogen receptor, progesterone receptor	4	46,XX,?der(3)t(3;11)(q21∼q23;q21∼q22),add(11)(q21)[12]	Not done	0.04 ± 0.00	0.00

^a^ Expressions of HMGA2 and PLAG1 were based on real-time PCR and the 2-ΔΔCq (Livak) method. The numbers are relative normalized expression ± standard error of the mean. The relative expression was calculated and set as 1 for human reference.

### Cytogenetics

**Figure 2:**
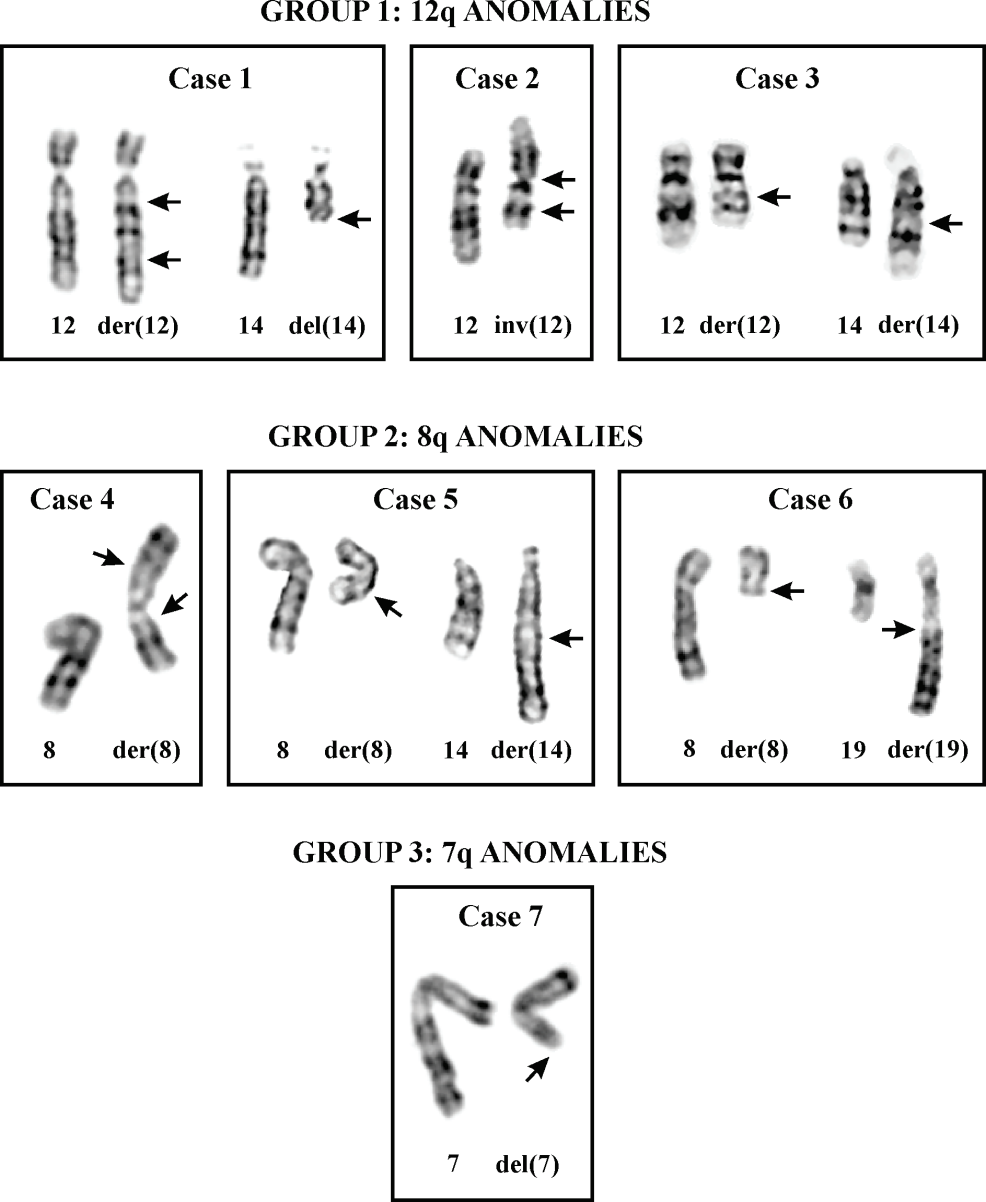
Cytogenetic analysis of leiomyomas of deep soft tissue Partial karyotypes of tumors of group 1 with 12q anomalies (cases 1-3), group 2 with 8q anomalies (cases 4-6), and group 3 with deletion 7q (case 7). The description of aberrations is given in Table 1. Arrows indicate breakpoints.

Based on the results of G-banding analysis, the leiomyomas were divided in 4 groups (Table 1, Figure 2): The first group consisted of leiomyomas with 12q rearrangements (cases 1-3). Case 1 had complex changes that included del(12)(q13q21) and ins(12;?)(q13;?) together with del(14)(q22) and monosomies for chromosomes 21 and 22. Case 2 had inv(12)(p11q15) as the sole abnormality. Case 3 had a t(12;14)(15;q24) translocation together with t(3;13)(p21;p13) and del(5)(q31q33). The second group consisted of leiomyomas carrying 8q rearrangements (cases 4-6). The tumors in cases 4, 5, and 6 had ins(8)(p23q12q22), t(8;14)(q13;q24), and t(8;19)(q12;q13) as sole cytogenetic abnormalities, respectively. The third group consisted of a single tumor with del(7)(q22) (case 7). In a subclone, del(14)(q24) was present in addition to deletion of 7q. The fourth group consisted of a tumor (case 8) with other cytogenetic aberrations: der(3)t(3;11)(q21∼23;q21∼22) and add(11)(q21).

### Fluorescence *in situ* hybridization (FISH) analysis

**Figure 3:**
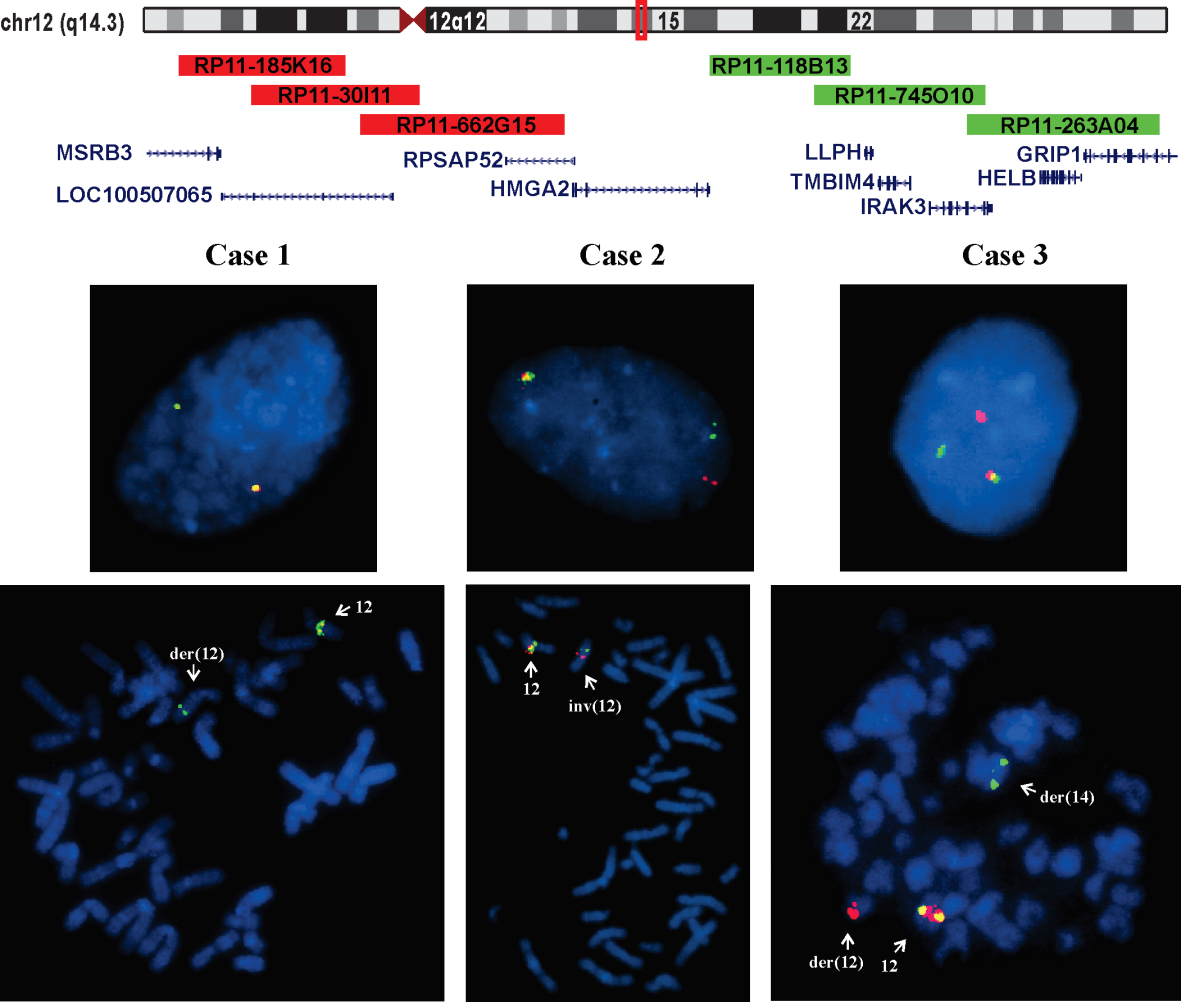
Interphase and metaphase FISH analyses of leiomyomas of deep soft tissue from group 1 with 12q anomalies Ideogram of chromosome 12 with the location of HMGA2 (red box) and the BACs used for FISH experiments are shown. The 5´-end of the probe (red signal) was constructed from a pool of the clones RP11-185K16, RP11-30I11, and RP11-662G15. The 3´-end of the probe (green signal) was constructed from a pool of the clones RP118B13, RP11-745O10, and RP11-263A04. In case 1, the analysis showed absence of the red signal (deletion of the 5´-end of the probe) in both metaphase spreads and interphase nuclei. In case 2, interphase FISH showed split between red and green signals (5´-end and 3´-end probes). The FISH on metaphase spreads showed that the green signal (3´-end of the probe) was moved to 12p. In case 3, interphase FISH showed split between red and green signals (5´-end and 3´-end probes). The FISH on metaphase spreads showed that the green signal (3´-end of the probe) had moved to 14q.

FISH with an *HMGA2* breakapart probe on interphase nuclei from cases 1-3 carrying 12q aberrations showed rearrangements of the *HMGA2* locus in 73 %, 43 %, and 98 % of the examined nuclei, respectively (Table 1, Figure 3). FISH on metaphase spreads was also possible (Figure 3). In case 1, the analysis showed deletion of the 5´-end of the probe (red signal). In case 2, the 3´-end of the probe (green signal) had moved to 12p. In case 3, the analysis showed that the 3´-end of the probe (green signal) had moved to 14q.

**Figure 4:**
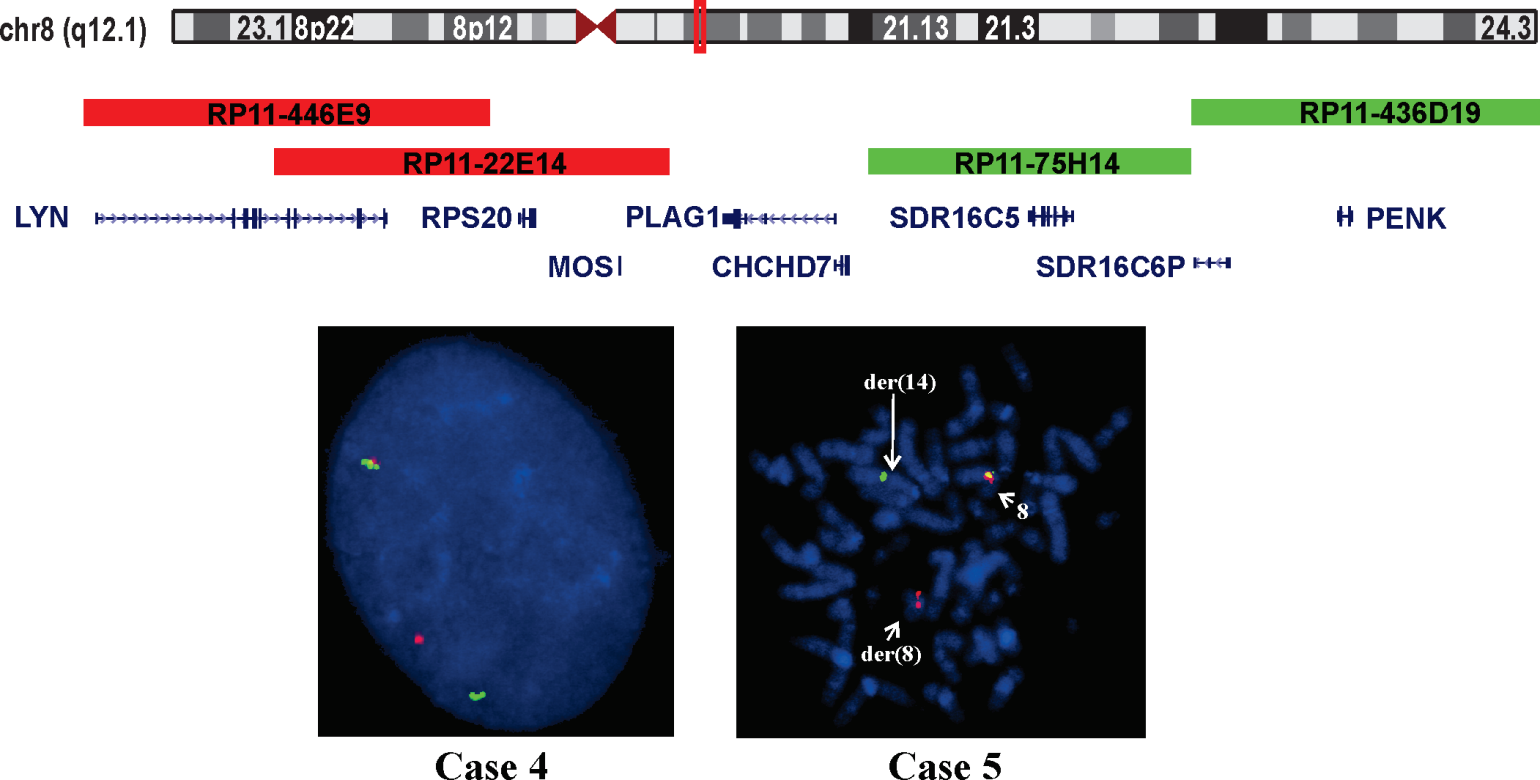
Interphase and metaphase FISH analyses of leiomyomas of deep soft tissue from group 2 with 8q anomalies Ideogram of chromosome 8 with the location of PLAG1 (red box) and the BACs used for FISH experiments are shown. The proximal probe (centromeric, red signal) consisted of the clones RP11-446E9 and RP11-22E14. The distal probe (telomeric, green signal) consisted of the clones RP11-75H14 and RP11-436D19. In case 4, FISH on interphase nuclei showed split between the proximal and distal probes. In case 5, FISH on metaphase spread showed that the green signal (distal probe) was moved to der(14) whereas the red signal (proximal probe) remained on der(8).

FISH with a *PLAG1* breakapart probe on interphase nuclei from the tumors of cases 4 and 5, which carried an 8q aberrations, showed rearrangement of *PLAG1* in 75 % and 86 % of the examined nuclei, respectively (Table 1, Figure 4). FISH on metaphase spreads from case 5 showed that the 3´-end of the probe (green signal) had moved to der(14) whereas the 5´-end of the probe remained on der(8) (Figure 4).

FISH could not be performed on cells from cases 6, 7, and 8.

### Expression of *HMGA2* and *PLAG1*

Results of the expression analysis for genes *HMGA2* and *PLAG1* using real-time PCR and the 2^−ΔΔCq^ method are shown in Table 1. In cases 1 and 2, with 12q rearrangements, *HMGA2* expression was approximately 8 and 6 times, respectively, stronger than the expression of *PLAG1*. No difference between *HMGA2* and *PLAG1* expression was seen in case 3 which also had a 12q abnormality. In cases 4 and 6, with 8q rearrangements, *PLAG1* but not *HMGA2* was expressed. In case 7, with del(7)(q22), and in case 8, with other changes, the expression of *HMGA2* was very low. *PLAG1* was expressed in case 7 but not in case 8 (Table 1).

### Expression of *MED12* and mutations in exon 2 of *MED12*

**Figure 5:**
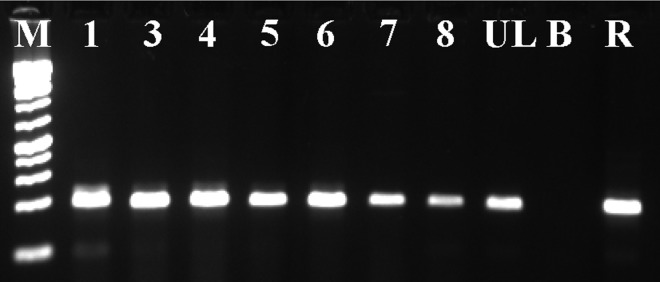
Expression of *MED12* in leiomyomas of deep soft tissue Gel electrophoresis of the RT-PCR amplified products. The primers MED12-Ex1-F/MED12-Ex3-R amplified a 163 bp cDNA fragment which contained part of exon 1, the entire exon 2, and part of exon 3 of the MED12 gene. All leiomyomas of deep soft tissue, except case 2, were examined (Lanes 1 and 3-8). M, 1 Kb DNA ladder (GeneRuler, Thermo Fisher Scientific). UL, uterine leiomyoma. B, Blank, water in cDNA synthesis. R, positive control, Human Universal Reference Total RNA (Clontech Laboratories, TaKaRa).

RT-PCR with the primers MED12-Ex1-F/MED12-Ex3-R amplified a 163 bp cDNA fragment in all examined leiomyomas of deep soft tissue (Figure 5). It contained part of exon 1, the entire exon 2, and part of exon 3 of the *MED12* gene suggesting that *MED12* was expressed. Sequencing of the PCR product did not show any mutation in the amplified cDNA fragment of *MED12* (data not shown).

### RNA-sequencing and verification of fusions

**Figure 6:**
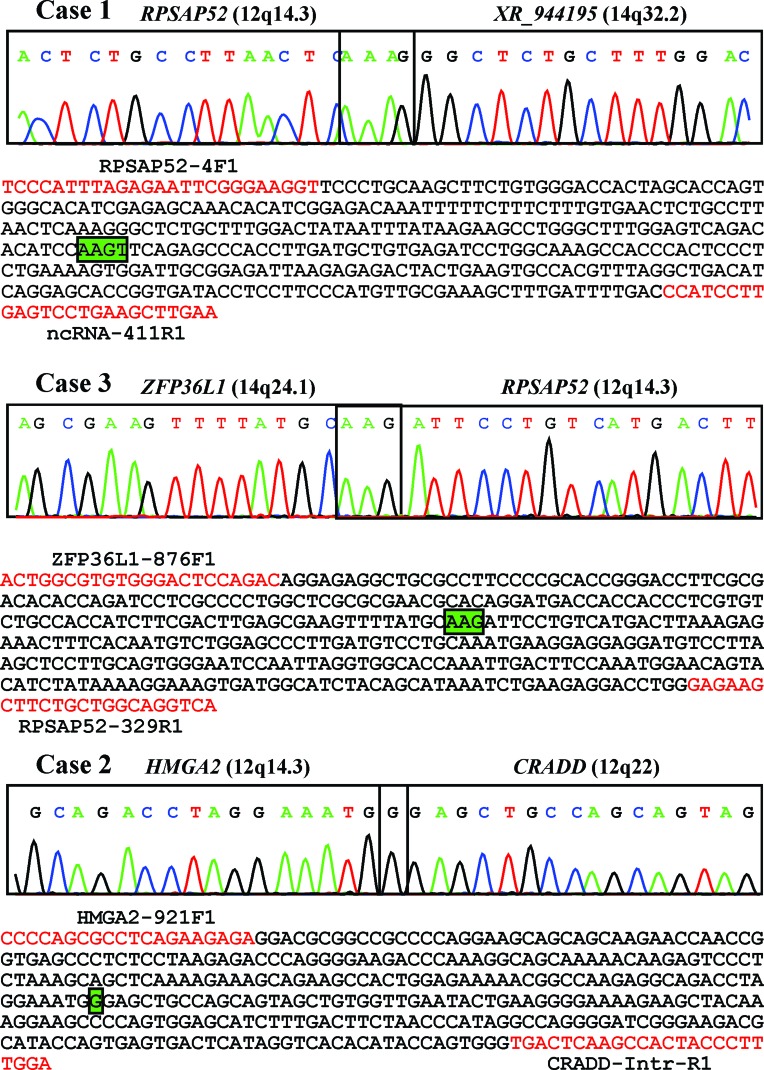
RT-PCR results on fusion genes related to 12q*/HMGA2* in cases 1, 3, and 2 The amplified cDNA fragments were direct sequenced. The partial chromatograms, which are shown, contain the fusion points. The chromosome locations of the fusion partners are shown in parentheses. The amplified cDNA sequences are also shown. Primers are in red. Common nucleotides found at the fusion points are shown in green boxes.

RNA-sequencing was performed for cases 1 and 3 in order to find fusion genes related to 12q/*HMGA2*. In case 1, a fusion of the ribosomal protein SA pseudogene 52 (*RPSAP52*) with the sequence with accession number XR_944195 was found (Figure 6). The *RPSAP52* pseudogene (accession number NR_026825.2, https://www.ncbi.nlm.nih.gov/nuccore/NR_026825?report=GenBank) maps on 12q14.3, next to *HMGA2*, and has two exons (https://www.ncbi.nlm.nih.gov/gene/204010). Exon 1 of *RPSAP52* is within intron 1 of *HMGA2*. The sequence with accession number XR_944195 (https://www.ncbi.nlm.nih.gov/nuccore/XR_944195) is a long non-coding RNA which maps on chromosome subband 14q32.2. RT-PCR followed by Sanger sequencing verified the presence of the *RPSAP52-XR_044195* fusion transcript in the tumor.

In case 3, the analysis detected a fusion between a sequence from *RPSAP52* and the *ZFP36L1* gene (ZFP36 ring finger protein like 1) which maps on chromosome subband 14q24.1 (Figure 6). RT-PCR followed by Sanger sequencing verified the presence of the *ZFP36L1-RPSAP52* fusion transcript.

In case 2, 3´-RACE amplified a single fragment (Figure 6). Sanger sequencing showed that it was a chimeric cDNA in which exon 3 of *HMGA2* was fused to a sequence in intron 1 of the *CRADD* gene from 12q22 (Figure 6). PCR with *HMGA2* forward and *CRADD* reverse primers amplified a cDNA fragment which by direct sequencing was shown to contain the same fusion point as the 3´-RACE amplified fragment (Figure 6).

## DISCUSSION

The presented data show that leiomyomas of deep soft tissue are genetically heterogenous and may arise through various tumorigenic pathways, most of which are already known from studies of uterine leiomyomas. Three out of the eight tumors had 12q aberrations (cases 1-3; Table 1). The translocation t(12;14)(q15;q24) found in case 3 is similar to that found in 20 % of karyotypically abnormal uterine leiomyomas [8, 9]. Complex karyotypes with 12q14∼15 and 14q22 rearrangements (case 3) were also reported in uterine leiomyomas [9]. In case 2, a pericentric inversion, inv(12)(p11q15), was found as the sole chromosomal abnormality. Again, similar inversions of chromosome 12 were also seen in uterine leiomyomas [9]. In addition, a leiomyoma of the vulva was reported to have an inv(12)(p12q13∼14) as the sole karyotypic change [10]. In FISH experiments, deletion of the 5´-end of the *HMGA2* probe (case 1) and moving of the 3´-end of the probe to 12p (case 2) or 14q (case 3) indicated that the rearrangements of 12q14∼15 targeted the *HMGA2* locus. The molecular analyses verified the FISH results. Thus, in case 2, the consequence of inv(12) was fusion of exon 3 of *HMGA2* with a sequence in intron 1 of the *CRADD* gene located in 12q22. The *HMGA2-CRADD* fusion transcript would code for a putative protein containing amino acid residues 1-83 of HMGA2 (accession number NP_003474.1) corresponding to exons 1-3 of the gene and 10 amino acid residues encoded by *CRADD* (ELPAVAVVEY). This pattern is similar to the rearrangements of *HMGA2* found in other benign connective tissue tumors, i.e., disruption of the *HMGA2* locus leaves intact exons 1-3 which encode the AT-hook domains and separates them from the 3´-terminal part of the gene [11, 12]. In uterine leiomyomas, *HMGA2* fusion transcripts were reported with various sequences including *COX6C* (8q22.2), *ALDH2* (12q24.12), *CCNB1IP1* (14q11.2), *RAD51B* (14q24.1), and RTVL-H 3_ LTR (21q21.2) [13]. However, the 12q14∼15 rearrangements, including t(12;14)(q15;q23∼24), were found to map predominantly to the 5´-region of *HMGA2* [14, 15]. The pattern of rearrangements suggests that the primary effect is dysregulated expression of *HMGA2*, most often by translocation of a chromosome 14 sequence upstream of the 5´-end of *HMGA2* [14, 15]. The chromosome aberration either separates from the exons a regulatory element that ordinarily negatively regulates *HMGA2* expression, or places a foreign regulatory element near *HMGA2* thus overcoming the silencing of this gene observed in normal adult tissues. Such regulatory elements might include an enhancer of gene expression. The paradigm for this mechanism is the overexpression of the *MYC* gene (8q24) as a result of the reciprocal translocations between chromosome 8 and chromosomes 2, 14, and 22 that harbor immunoglobulin loci expressed in B-lineage lymphatic cells [8].

In cases 1 and 3 (Table 1), which were subjected to RNA-sequencing, fusion transcripts of *RPSAP52*, the ribosomal protein SA pseudogene 52, were found (Reference Sequence: NR_026825.2, https://www.ncbi.nlm.nih.gov/gene/204010). *RPSAP52* has two exons: Exon 1 is located within intron 1 of *HMGA2* whereas exon 2 is located 65 kbp upstream of exon 1 of *HMGA2*. In case 1, exon 1 of *RPSAP52* was fused with the noncoding RNA with accession number XR_944195 from chromosome band 14q32.2. *RPSAP52* was the 5´-end partner in the fusion transcript. In case 3, exon 2 of *RPSAP52* was fused with *ZFP36L1* which maps on 14q24.1. *RPSAP52* was the 3´-end partner in the *ZFP36L1-RPSAP52* fusion transcript. The findings indicate that the breakpoints lie upstream of the 5´-end of *HMGA2* and that, in leiomyomas of deep soft tissue with rearrangements of 12q13∼15, fusion of *HMGA2* is not the only molecular mechanism whereby abnormal expression of this gene can be achieved. Thus, the differences in relative normalized expression of *HMGA2* seen in Table 1 could reflect differences in molecular mechanisms behind the abnormal expression of *HMGA2*: In case 2, the expression was the result of an *HMGA2-CRADD* fusion transcript that disrupted *HMGA2* but left intact exons 1-3 coding for the AT-hook domains and separated them from the 3´-untranslated region of the gene (3´-UTR) [11]. The 3´-UTR of *HMGA2* was shown to regulate the transcription of the *HMGA2* gene [16, 17]. In cases 1 and 3, *HMGA2* expression probably was the result of chromosome 14 sequences being moved upstream of *HMGA2* by the chromosome rearrangements. Thus, different regulatory elements could be placed near *HMGA2*. In the leiomyomas of deep soft tissue without 12q13∼15 rearrangements, *HMGA2* expression was zero (cases 4 and 6 with 8q rearrangements) or very low (cases 7 and 8). Although the number of leiomyomas of deep soft tissue so far studied is still very low, the findings with regard to 12q13∼15 aberrations/expression of *HMGA2* seem to be similar to those made in uterine leiomyomas [14, 15].

Three out of eight tumors had aberrations of chromosome bands 8q12∼13 (cases 4-6; Table 1). FISH examination showed that *PLAG1* was split in cases 4 and 5. Moreover, expression analysis of cases 4 and 6 showed that *PLAG1* was expressed whereas expression of *HMGA2* was absent. Taking the FISH and expression analyses together, *PLAG1* seems to be targeted by 8q aberrations in leiomyomas of deep soft tissue. The difference in *PLAG1* expression between cases 4 and 6 could be the result of different regulatory elements being placed near *PLAG1* by the different aberrations. Chromosome rearrangements of 8q11∼13 but without concomitant involvement of 12q13∼15 (where *HMGA2* maps) or 6p21 (where the *HMGA1* gene is situated) was reported in six uterine leiomyomas, one leiomyoma of the vagina, and one intraabdominal leiomyoma [9]. However, in the leiomyoma of the vulva with karyotype 46,XX,t(7;8)(p13;q11.2), the *PLAG1* gene was not altered by the translocation [18].

Recently, uterine leiomyomas with *HMGA2* aberrations were shown to display highly significant up-regulation of *PLAG1* [19, 20]. Both the leiomyomas with 12q14∼15 rearrangements/expression of *HMGA2* (cases 1-3; Table 1) and case 7 with del(7)(q22) expressed *PLAG1* (Table 1). No expression of *PLAG1* was seen in case 8 which had neither 12q13∼15 change nor del(7)(q22) (Table 1).

The terminal deletion del(7)(q22) found in case 7 (Table 1) was also reported in uterine leiomyomas [9]. However, an interstitial deletion of chromosome 7, del(7)(q22q32), is much more frequent as it occurs in 20% of karyotypically abnormal uterine leiomyomas [8, 9]. The pathogenetically important region in 7q22 was in one study narrowed down to a 500 kb gene-dense area [21]. Furthermore, in two uterine leiomyomas, one with a pericentric and the other with a paracentric inversion of chromosome 7, the inversions targeted the cut-like homeobox1 (*CUX1*) gene on chromosomal band 7q22.1 [22]. Whether this represents the whole pathogenetic story behind 7q-deletions in leiomyomas, remains a moot point.

In case 8, rearrangements of chromosome bands 3q21∼23 and 11q21∼22 were found. Involvement of these bands was previously reported also in uterine leiomyomas. Deletion of 3q21 was reported as the sole anomaly in a uterine leiomyoma [23], and Dal Cin et al [24] reported an interstitial deletion of the long arm of chromosome 3 as the sole abnormality in another three such tumors. Although there was cytogenetic heterogeneity of the deleted 3q segment (q13.3-q27 in case 1, q12-q24 in case 2, and q21-q27 in case 3), the authors concluded that the involvement of 3q was significant enough to define a new cytogenetic subgroup [24]. Indeed, a more recent study described two more uterine leiomyomas with deletions of the long arm of chromosome 3 [25]. Both led to loss of the *MED12L* gene (3q25.1) which shows strong similarity with *MED12*. Finally, the translocations t(5;11)(q13;q21) and t(6;11)(p23;q21) were reported in two uterine leiomyomas as the sole cytogenetic abnormality [26, 27]. The molecular consequences behind rearrangements of 3q21 and 11q21 remain unknown.

A number of recent studies have reported mutations in exon 2 of the *MED12* gene in most uterine leiomyomas [28]. Mutations of *MED12* were found in leiomyomas with a normal karyotype, with deletions or rearrangements of the long arm of chromosome 7 as sole anomaly, and with 6p21∼23 abnormalities leading to *HMGA1* rearrangement/overexpression, and it was concluded that they precede the chromosomal aberrations [29]. On the other hand, mutations of *MED12* have not been detected in uterine leiomyomas with 12q14∼15 rearrangements resulting in overexpression of *HMGA2* [29, 30]. In a recent study, *MED12* mutations were also found in 10 out of 29 (34%) cases of leiomyoma/leiomyomatosis in pelvic/retroperitoneal sites [5]. In another study, Ravegnini et al [31] found *MED12* mutations in 3 of 19 (16%) extrauterine leiomyomas (one each from the ovary, kidney, and retroperitoneum). In contrast, none of the 42 extrauterine leiomyomas had *MED12* mutations in exon 2 in the study published by Matsubara et al [32]. In the present study, no mutations in exon 2 of *MED12* were found in eight leiomyomas of deep soft tissue. Thus, the role of exon 2 *MED12* mutations in the development of extrauterine leiomyomas, including leiomyomas of deep soft tissue, appears to be limited.

In a previous study [6], we reported the finding of a *KAT6B-KANSL1* fusion gene in a retroperitoneal leiomyoma with t(10;17)(q22;q21). Uterine leiomyomas with t(10;17) and disruption of the *KAT6B* gene were also described [33]. In another study [7], we found an *EWSR1-PBX3* fusion gene in a retroperitoneal leiomyoma carrying a t(9;22)(q33;q12) chromosome translocation. The present findings together with data on those two previously described tumors show that leiomyomas of deep soft tissue are genetically heterogeneous but with marked similarities to uterine leiomyomas.

## MATERIALS AND METHODS

### Patients

The material consisted of eight samples from tumors diagnosed as leiomyoma of deep soft tissue (Table 1), all surgically removed at The Norwegian Radium Hospital between 2006 and 2015. The study was approved by the Regional Committee for Medical and Health Research Ethics, South-East Norway (REK Sør-Øst;http://helseforskning.etikkom.no). Written informed consent was obtained from the patients. The consent included acceptance that the clinical details be published. The ethics committee's approval included a review of the consent procedure. All patient information has been de-identified.

### Chromosome banding analysis and FISH

Samples from the surgically removed tumors were received and analyzed cytogenetically as part of our diagnostic routine using standard techniques [34]. Chromosome preparations were G-banded using Wright's stain (Sigma-Aldrich; St Louis, MO, USA). The subsequent cytogenetic analysis and karyotype description followed The International System for Human Cytogenetic Nomenclature (ISCN) 2016 guidelines [35].

FISH analysis based on the karyotypic findings (see below) was performed on both interphase nuclei and metaphase plates. *HMGA2* BAC clones were retrieved from the Human genome high-resolution BAC re-arrayed clone set (the “32k set”; BACPAC Resources, http://bacpac.chori.org/pHumanMinSet.htm). Detailed information on the *HMGA2* BAC clones is given elsewhere [36]. A homemade breakapart *HMGA2* probe was used. The 5´-end of the probe (red signal) was constructed from a pool of the clones RP11-185K16, RP11-30I11, and RP11-662G15. The 3´-end of the probe (green signal) was constructed from a pool of the clones RP118B13, RP11-745O10, and RP11-263A04. All of them map to chromosome subband 12q14.3 and cover the *HMGA2* locus [36].

For the *PLAG1* locus, clones were based on the contigs used to construct the GRCh38 (hg38) genome assembly as well as the NCBI clone end mappings from the NCBI Clone DBdatabase (http://www.ncbi.nlm.nih.gov/clone/library/genomic/12/). A homemade breakapart *PLAG1* probe was used. The proximal or centromeric probe (red signal) consisted of the clones RP11-446E9 (accession number AC046176, position chr8:55870288-56054628) and RP11-22E14 (accession number AC083961, position chr8:55956731-56135453). The distal or telomeric probe (green signal) consisted of the clones RP11-75H14 (accession number AC103849, position chr8:56227167-56374265) and RP11-436D19 (accession number AC023464, position chr8: 56299162-56459550).

DNA was extracted, the probes were labelled with Fluorescein-12-dCTP (PerkinElmer, Boston, MA, USA) and Texas Red-5-dCTP (PerkinElmer) in order to obtain green and red signals, respectively, using the Abbott's nick translation kit (Des Plaines, IL, USA), and hybridized according to Abbott Molecular recommendations (http://www.abbottmolecular.com/home.html). Chromosome preparations were counterstained with 0.2 μg/ml DAPI and overlaid with a 24 × 50 mm^2^ coverslip. Fluorescent signals were captured and analyzed using the CytoVision system (Leica Biosystems, Newcastle, UK).

### RNA extraction and cDNA synthesis

Total RNA was extracted using miRNeasy Mini Kit according to the manufacturer's instructions (Qiagen Nordic, Oslo, Norway) from frozen and stored at −80°C tumor tissue adjacent to that used for cytogenetic analysis and histologic examination. No material for RNA extraction was available from case 5. The tissue was disrupted and homogenized in Qiazol Lysis Reagent (Qiagen) using 5 mm stainless steel beads and TissueLyser II (Qiagen). Subsequently, total RNA was purified using QIAcube (Qiagen). The RNA quality was evaluated using the Experion Automated Electrophoresis System (Bio-Rad Laboratories, Oslo, Norway).

One μg of total RNA was reverse-transcribed in a 20 μL reaction volume using iScript Advanced cDNA Synthesis Kit for RT-qPCR according to the manufacturer's instructions (Bio-Rad Laboratories, Oslo, Norway). The cDNA was diluted to 50 μl of which 1 μl was used as template in subsequent PCR assays.

### Expression analysis of HMGA2 and PLAG1

Real time PCR was carried out to determine the expression level of the *HMGA2* and *PLAG1* genes using TaqMan gene expression assays (Applied Biosystems, Foster City, CA, USA) Hs04397751_m1 (*HMGA2* exons 2-3 in sequence with accession number NM_00003483) and Hs00965049_g1 (*PLAG1* exons 4-5 in sequence with accession number NM_002655.2). The genes *ACTA2*, *DES*, and *CALD1* which code for aortic smooth muscle actin, desmin, and caldesmon 1, respectively, were used as endogenous controls for relative gene expression quantification. The assay for *ACTA2* was the Hs00426835_g1 which spans the boundary between exons 2 and 3 (accession number NM_001613.2). The assay for *DES* was the Hs00157258_m1 which spans the boundary between exons 6 and 7 (accession number NM_001927.3). The assay for *CALD1* was the Hs00921982_m1 which spans the boundary between exons 12 and 13 (accession number NM_004342.6).

Human Universal Reference Total RNA was used as control (Clontech Laboratories, TaKaRa Bio Group, France). According to the company's information, it is a mixture of total RNAs from a collection of adult human tissues chosen to represent a broad range of expressed genes. Both male and female donors are represented.

Four replicates of each sample and endogenous control were used. The 20 μL reaction volume contained 1x TaqMan Universal Mix, 1x 20x TaqMan Gene Expression Mix, and 2 μL cDNA (40 ng equivalent of RNA). Real time PCR was run on a CFX96 Touch™ Real-Time PCR Detection System (Bio-Rad Laboratories). The thermal cycling included an initial step at 50°C for 2 min, followed by 10 min at 95°C and 40 cycles of 15 sec at 95°C, and 1 min at 60°C. The data were analyzed using the Bio-Rad CFX Manager 3.1 Software (Bio-Rad Laboratories). The 2^−ΔΔCq^ (Livak) method for relative gene expression was used [37]. Expression of the different transcripts was normalized to *ACTA2*, *DES*, and *CALD1* expression before the relative expression was calculated and set as 1 for human reference (Table 1).

### RT-PCR analysis of the MED12 gene

The primers MED12-Ex1-F (5′-TTA CCC TCA GGA CCC CAA ACA G-3′) and MED12-Ex3-R (5′-TGC AAT AAT GCT GCT GAA GTT GG-3′) were used for assessment of the expression of *MED12* and detection of possible mutations in exon 2 of *MED12*. Detailed information about the assay is given elsewhere [6]. A uterine leiomyoma with t(12;14)(q14∼15;q23∼24) and the Human Universal Reference Total RNA were used as controls.

### 3´- Rapid amplification of cDNA ends (3´- RACE)

The 3′-RACE methodology used was described in detail elsewhere [36].

### RNA-sequencing

Three μg of total RNA were sent for high-throughput paired-end RNA-sequencing at the Norwegian Sequencing Centre, Ulleval Hospital (http://www.sequencing.uio.no/). Detailed information about the RNA sequencing is given elsewhere [6, 38]. The softwares deFuse, FusionCatcher, and TopHat-Fusion were used for the discovery of fusion transcripts [39–41].

### RT-PCR analyses for verification of fusion transcripts

For reverse transcriptase-Polymerase Chain Reaction (RT-PCR), the 25 μL PCR volume contained 12.5 μL Premix Ex Taq™ DNA Polymerase Hot Start Version Taq (Takara Bio Europe/SAS, Saint-Germain-en-Laye, France), cDNA, and 0.4 μM of each of the forward and reverse primers. The PCR was run on a C-1000 Thermal cycler (Bio-Rad Laboratories) with an initial denaturation at 94°C for 30 sec, followed by 35 cycles of 7 sec at 98°C, 30 sec at 60°C, 1 min at 72°C, and a final extension for 5 min at 72°C. The primer combinations were the following: In case 1, RPSAP52-4F1 (TCCCATTTAGAGAATTCGGGAAGGT) and ncRNA-411R1 (TTCAAGCTTCAGGACTCAAGGATGG); in case 2, HMGA2-921F1 (CCCCAGCGCCTCAGAAGAGA) and CRADD-Intr-R1 (TCCAAAGGGTAGTGGCTTGAGTCA); and in case 3, ZFP36L1-876F1 (ACTGGCGTGTGGGACTCCAGAC) and RPSAP52-329R1 (TGACCTGCCAGCAGAAGCTTCTC). Three μL of the PCR products were stained with GelRed (Biotium, Hayward, CA, USA), analyzed by electrophoresis through 1.0 % agarose gel, and photographed. The remaining PCR products were purified using the Qiaquick PCR purification kit (Qiagen) or the QIAquick Gel Extraction Kit (Qiagen, Hilden, Germany) and direct sequenced using the dideoxy procedure with the ABI Prism BigDye terminator v1.1 cycle sequencing kit (ThermoFisher Scientific, Waltman, MA, USA) on the Applied Biosystems Model 3500 Genetic Analyzer sequencing system. BLAST and BLAT softwares (http://www.ncbi.nlm.nih.gov/BLAST/, http://genome.ucsc.edu/cgi-bin/hgBlat?command=start) were used for computer analysis of sequence data.
